# Pulmonary ACE2 expression in neonatal and adult rats

**DOI:** 10.1002/2211-5463.13232

**Published:** 2021-06-30

**Authors:** Depeng Zhao, Xueyu Chen, Dongshan Han, Junyan Zhong, Su‐E Zhang, Chuanzhong Yang

**Affiliations:** ^1^ Department of Reproductive Medicine Affiliated Shenzhen Maternity and Child Healthcare Hospital Southern Medical University Shenzhen China; ^2^ Laboratory of Neonatology Department of Neonatology Affiliated Shenzhen Maternity and Child Healthcare Hospital Southern Medical University Shenzhen China; ^3^ Department of Neonatology Shenzhen Maternity and Child Healthcare Hospital The First School of Clinical Medicine Southern Medical University Shenzhen China

**Keywords:** ACE2, COVID‐19, expression, newborn, SARS‐CoV‐2

## Abstract

Children show a distinct presentation of COVID‐19, characterized by a lower incidence and mild phenotype, but the reason for this is still unknown. The angiotensin‐converting enzyme 2 (ACE2) functions as the primary cell entry receptor for Severe acute respiratory syndrome coronavirus 2 (SARS‐CoV‐2) and is thought to cause distinct clinical features between children and old people. The primary purpose of this study was to determine whether differences exist in the level of expression and distribution of ACE2 between neonatal and adult rat lungs. The lung tissues from rats of various ages were used to investigate the expression patterns of ACE2. Western blot, immunohistochemistry, and immunofluorescence were used to quantify or identify the localization of ACE2 in rat lungs. ACE2 was homogenously expressed in fewer alveolar type II (AT2) cells in the neonatal lung, with no polarization to the alveolar space and additional expression in pulmonary endothelium when compared to adult rat lungs. These findings suggest that the patterns of ACE2 distribution and cellular localization in rat lungs change with age.

## Graphical abstract

Here, we examined whether differences exist in the level of expression and distribution of ACE2 between neonatal and adult rat lungs. We found that ACE2 was homogenously expressed in fewer alveolar type II cells in the neonatal lung, with no polarization to the alveolar space and additional expression in pulmonary endothelium when compared to the adult rat lung.

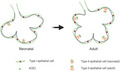

**Abbreviations**
ACE2: Angiotensin‐converting enzyme 2
AT2: Alveolar type II
COVID‐19: Coronavirus disease 2019
IFN: Interferon
SARS‐CoV‐2: Severe acute respiratory syndrome coronavirus 2
SPC: Prosurfactant protein C

The coronavirus disease 2019 (COVID‐19) pandemic is caused by severe acute respiratory syndrome coronavirus 2 (SARS‐CoV‐2) [1]. While SARS‐COV‐2 infections appear in individuals of any age, the severity and clinical presentation of COVID‐19 are quite different between children and adults [2]. Generally, severe cases of COVID‐19 are less likely to occur in children compared with their adult counterparts [3, 4, 5]. Several theories are proposed to explain why COVID‐19 appears to be mild in children, including differences in the composition and functional responsiveness of the immune system [4], the coexisting viruses in the airway of children [6], and the expression of receptors for the virus [7]. In addition to the severity of COVID‐19, the clinical presentations of COVID‐19 are also dissimilar in children from those in adults; children with COVID‐19 show a higher rate of cardiovascular involvement [8, 9]. The reasons for different clinical symptoms of COVID‐19 between children and adults remain elusive [6]. Several studies indicate that age is an important factor that influences ACE2 expression in the lungs [10, 11]. In addition, the cellular localization of ACE2 at the apical membrane of alveolar epithelia facilitates the binding and cell entry of the SARS‐CoV‐2 virus [12, 13]. However, there is a paucity of firm evidence to confirm these assumptions [14]. In this study, we hypothesized that the ACE2 expression at the cellular level may be different between children and adults, resulting in dissimilarity in clinical signs. To test this hypothesis, the expression profiles of ACE2 in rats’ lung tissue from various ages were compared. Therefore, the primary purpose of this study was to determine whether in rats differences existed in the level of expression and distribution of ACE2 between neonatal and adult lungs.

## Materials and methods

### Animals

All animal experiments were approved by the Institutional Animal Care and Use Committee of Shenzhen Institutes of Advanced Technology Chinese Academy of Sciences. Newborn pups from four pregnant Wistar rats were randomized to 4 groups (*N* = 8) and sacrificed at postnatal days 3, 30, 60, and adult (3 months old). Rats were anesthetized at the designed day by intraperitoneal injection of pentobarbital (40 mg/Kg). Blood was withdrawn from the abdominal artery to sacrifice the animals. Hereafter, lungs were either snap‐frozen in liquid nitrogen for Western blot (*N* = 4) or fixed in situ in 4% paraformaldehyde (PFA) under a pressure of 27 cm H2O (*N* = 4), as described previously [15].

### Immunohistochemistry

The lung was fixed in 4% PFA overnight and embedded in paraffin. Lung tissues were incubated with an anti‐ACE2 antibody (ab15348; Abcam, diluted in 1 : 500), followed by a horseradish peroxidase (HRP)‐conjugated goat anti‐rabbit antibody (ab6721; Abcam, Waltham, MA, USA, diluted in 1 : 1000). The color was developed by the chromogenic substrate NovaRed (Vector, Burlingame, CA, USA). The tissues were photographed by Olympus CX43 (Tokyo, Japan) and WZ Camera S50 (Shenzhen, China).

### Western blot

Approximate 50 mg lung tissue was homogenized by an Ultra‐Turrax T10 tissue homogenizer (IKA, Germany) for 20 s at full speed in 600ul RIPA (89 900, Thermo Scientific, Waltham, MA, USA) with Protease Inhibitor Cocktail (87 786, Thermo Scientific). The homogenate was incubated on ice for 30 mins and centrifuged at 18 400 ***g*** for 20 min. The supernatant was aliquoted and stored at −80 degree. Before loaded to the gel, the total protein concentration was quantified using a commercial bicinchoninic acid (BCA) kit (PC0021; Solarbio, China) and measured at 562 nm. A total amount of 30 ug protein was loaded to SDS/PAGE (10% separating gel, 5% stacking gel) and blotted onto the polyvinylidene fluoride (PVDF) membrane (Millipore, USA). The blot was cut and incubated with an anti‐ACE2 antibody (ab15348, Abcam, 1 : 1000) or anti‐β‐actin antibody (AM4302, Invitrogen, Waltham, MA, USA, 1 : 5000) overnight. After washing with phosphate‐buffered saline with Tween 20 (PBST), the blots were subjected to an HRP‐conjugated goat anti‐rabbit antibody (SA00001‐2; Proteintech, Rosemont, IL, USA, diluted in 1 : 5000) for 30 mins and visualized with ECL substrates (32106, Pierce) on BioDocAnalyze (BDA) digital (Analytik Jena, Germany). The bands were quantified with imagej (NIH, USA) as others had described [16].

### Immunofluorescence

To co‐locate ACE2 and alveolar type 2 cells (AT2), paraffin‐embedded lung tissue was sectioned continuously to acquire the same tissue for separate staining of ACE2 and SPC. The same lung sections were, respectively, incubated with anti‐ACE2 antibody (ab15348, 1 : 100) and anti‐SPC antibody (Prosurfactant protein C, ab90716, Abcam, diluted in 1 : 300) overnight at 4°. Lung sections were later incubated with Alexa Fluor 488‐conjugated donkey anti‐rabbit antibody (A‐21206, Invitrogen, Waltham, MA, USA diluted in 1 : 800) or Alexa Fluor plus 555‐conjugated donkey anti‐rabbit antibody (A32732, gen, diluted in 1 : 800) in the dark for 2 h. The tissues were covered with Prolong Gold antifade reagent with DAPI (8961, Cell Signaling Technology, Danvers, MA, USA) and cured in the dark for 24 h and then sealed with nail polish. The tissues were photographed by Olympus IX73 and cellSens Standard 3 (Tokyo, Japan).

### Statistical analysis

Differences between experimental groups in the quantification of blots were analyzed by one‐way ANOVA, followed by Dunnett test. For statistical analysis, the graphpad prism version 7 software package was used (San Diego, CA, USA). A *P* < 0.05 was considered statistically significant.

## Results

### Expression profile of ACE2 in neonatal and adult rat lungs

**Fig. 1:**
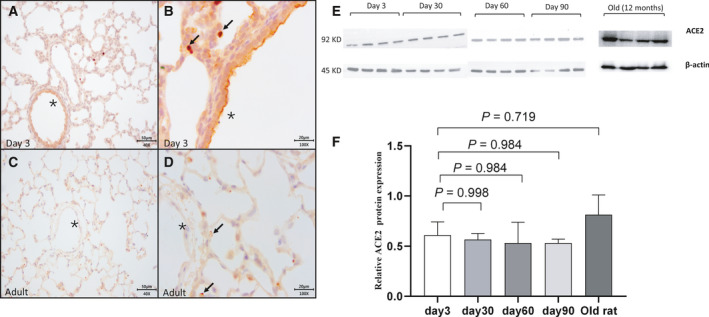
Immunohistochemistry staining and pulmonary protein level of ACE2 in rat’s lung tissues at different ages (Fig. 1A,B for neonatal lungs, Fig. 1C,D for adult lungs). In neonates, cells stained positively for ACE2 were few in neonatal lungs (Fig. 1B, arrows), while ACE2 was stained extensively in the adult lung (Fig. 1C) with a special profile of polarization to the alveolar space (Fig. 1D, arrows). Pulmonary endothelium was stained positive for ACE2 in neonatal rather than adult lung (Fig. 1B,D, stars). Scale bars, 50 μm in panel A and C; 20 μm in panel B and D. A total amount of 30 μg protein was loaded to the gel, and β‐actin was used as a reference (Fig. 1E,F *N* = 4). Each group was compared with the group of Day 3 using one‐way ANOVA, followed by Dunnett multiple comparisons test. (Fig. 1F). The bars were presented as mean with standard error of the mean (SEM).

In the neonatal lung, ACE2 was positive in a limited number of cells (Fig. 1A), mainly in the alveolar (Fig. 1B, arrows) and endothelium (Fig. 1A,B, star). Unlike neonates, ACE2 in the adult lung was absent in pulmonary endothelium (Fig. 1C,D, star) and exclusively expressed in alveolar cells (Fig. 1D, arrows). Furthermore, ACE2 was homogenously stained in cytoplasm or nucleus in the neonatal lung (Fig. 1B, arrows), while it was polarized to the alveolar space in adult alveoli (Fig. 1D, arrows).

### The protein level of ACE2 in the lung of rats from various age

As shown in Fig. 1, the protein levels of ACE2 in the rat lungs seemed to be stable across ages (Fig. 1E). ACE2 expression was relatively higher in old rats (at age of 12 months) but did not reach a significant difference (Fig. 1F). The quantification of each blot is listed in Table S1.

### Co‐localization of ACE2 and SPC in neonatal and adult rat lungs

**Fig. 2:**
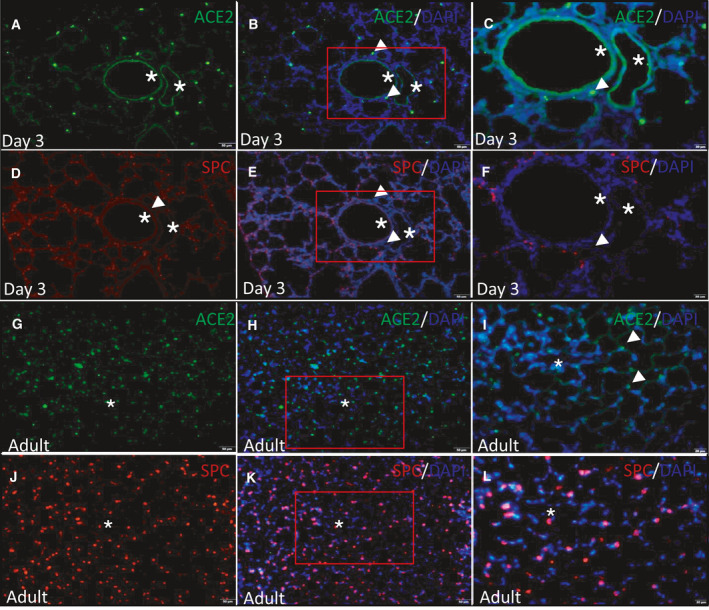
Co‐location of ACE2 and AT2 in neonatal (Fig. 2A–F) and adult (Fig. 2G–L) rat lung tissue. In the neonatal lung, ACE2 (green)‐positive cells were partly overlapped with SPC (red)‐positive cells (Fig. 2B,E, arrows); the endothelium was stained positive for ACE2 (Fig. 2C, stars). In the adult lung, ACE2 (green) was stained extensively in the adult lung, as well as SPC (red, Fig. 2H,K), the endothelium was stained negative for ACE2 (Fig. 2H,I, stars). The nucleus was stained with DAPI (blue). Fig. 2B,E is the same lung tissue acquired by continuously sectioning, the same with Fig. 2H,K. Fig. 2C,F,I, and L is the magnification of the red square areas in Fig. 2B,E,H, and K, respectively. Original magnification C, F, I, and L, ◊400; the rest, ◊200. Scale bars, 50 μm in panel A, B, D, E, G, H, J, and K; 20 μm in panel C, F, I, and L.

Like the finding from immunohistochemistry, ACE2 was expressed in both alveolar and endothelium in the neonatal lung (green, Fig. 2A–C). However, when compared with the same lung tissue stained for SPC (red, Fig. 2D–F), we found ACE2 in alveolar was also expressed in SPC‐negative cells (Fig. 2C, and 2F, arrows), and only a small number of SPC‐positive cells expressed ACE2 (Fig. 2C,F).

Unlike neonatal rats, the expression of ACE2 was absent in endothelium from adult lung (Fig. 2G–I, star). A relatively large proportion of SPC‐positive cells were stained with ACE2 in the adult lung when compared to the neonatal lung (Fig. 2J–L). Furthermore, the polarization of ACE2 to the alveolar space in the adult lung was further confirmed (Fig. 2I, arrows). Similar to neonatal rats, ACE2 was also expressed in SPC‐negative cells (Fig. 2I,L).

## Discussion

While ACE2 is recognized as the key cell entry receptor for SARS‐CoV‐2 [17], little is known on the patterns of ACE2 distribution and cellular localization. In this study, we demonstrated the pulmonary ACE2 is distinctly distributed and localized between neonatal and adult rats.

First, the ACE2‐positive cells were scattered in the neonatal rat lungs, while they were extensively detected in adult rat lungs (Fig. 1), We also found that neonatal pups had fewer AT2 cells (SPC‐positive) compared with adult rats (Fig. 2), which was consistent with the ACE2 expression in human lungs in other studies [7, 18, 19]. However, Zhang *et al*. reported ACE2+ cells were extensively stained in lung biopsies from young patients while few were observed in elder patients with COVID‐19 [14]. This discrepancy might be mainly attributed to a different species studied. Moreover, the inflammatory milieu in the lung of COVID‐19 patients may alter the physiological expression of the ACE2. Therefore, the patterns of ACE2 expression in a healthy animal model may not represent what is observed in COVID‐19 patients. In this study, a particular expression pattern of ACE2 characterized by polarization to the alveolar space was only observed in adults rather than the neonatal lung, which was also demonstrated in adult mice by Yee *et al*. [19]. Several studies reported that ACE2 protein is low in lung tissue [20, 21]. Since the alveolar epithelia are well polarized in lungs [22], the cellular distribution of ACE2 protein is significant to affect the pathogenesis of COVID‐19. This study showed that the ACE2 protein is less intensely located at apical membranes of alveolar epithelia in neonatal rat lungs compared with adult ones (Fig. 1). However, it is unclear whether this specific localization pattern of alveolar epithelial ACE2 exists in human lungs.

Second, we found pulmonary vascular endothelium was positive for ACE2 staining in neonatal lungs whereas it was negative in adult lungs (Fig. 2). However, Ma *et al*. found the aorta from aged primates (18–21 years old) expressed a higher amount of ACE2 at mRNA level compared with the aorta from young primates (4–6 years old) [18]. Similarly, Xie *et al*. and Hamming *et al*. found ACE2 expression was also present in the endothelium from adult rat lungs and major human organs including lung [23, 24]. However, McCracken *et al*. recently challenged these findings and raised concern on the evidence of ACE2 expression in human endothelial cells [25]. This difference might be attributed to the experimental methods. While Xie *et al*. and Hamming *et al*. used immunohistochemistry and immunofluorescence‐based assays, McCracken *et al*. employed an RNA‐seq approach [18]. Importantly, the age‐dependent expression of ACE2 in endothelial cells remains to be elucidated in COVID‐19 patients.

Third, by comparing the localization of ACE2‐positive cells and SPC‐positive cells, we found that other cells besides AT2 expressed ACE2 in both neonates and adults (Fig. 2), which was also confirmed in other studies [23, 24]. Ma *et al*. found ACE2 was expressed in AT2, AT1, ciliated cells, fibroblasts, pericytes, and immune cells including alveolar macrophages, T cells, and B cells in the lung [18]. This might explain why we found an overall stable expression of ACE2 protein in lungs from different ages studied, whereas Xie *et al*. reported pulmonary ACE2 protein decreased with age in adult rats, especially in the elder [24], indicating a dynamic protein profile of pulmonary ACE2 in a lifespan.

This study has several limitations. First, we failed to visualize the ACE2 and SPC on the same tissue slide by double staining due to the lack of appropriate primary antibodies. To quantify the expression of ACE2 in AT2 cells, continuous sections of lung tissues were used to co‐locate the positive signal of ACE2 and AT2 cells. Second, the mechanisms underlying the differential pattern of ACE2 expression between neonates and adults were not clarified in this study, which may provide insight into the therapeutic target for COVID‐19 treatment. Third, the function and structure of ACE2 vary to some extent between upper and lower airway and among different species [21]. However, the most significant one is we did not investigate the direct immune response to the virus, especially the interferon (IFN) since several studies had reported the virus proteins suppresses INF response [26, 27]. Last but not the least, the quantification of the blots might not precisely reveal the ACE2 protein level since concerns have been raised regarding the accuracy of quantification of the blots [28].

In summary, this study demonstrates that the expression pattern of ACE2 is different in the neonatal rat lungs compared with the adult counterparts. The expression in neonatal rat lungs is characterized by homogenous expression in fewer AT2 cells, no polarization to the alveolar space, and expression in pulmonary endothelium. Our findings suggest that the distribution and localization of ACE2 at cellular level in rat lungs vary with aging.

## Conflict of interest

The authors declare no conflict of interest.

## Author contributions

DZ, XC, and CY designed the experiment. XC, DZ, and JZ conducted experiments. XC, DH, and SZ performed data analysis. DZ, XC, and CY wrote or contributed to the manuscript.

## Supporting information


**Table S1**. Quantification of each blot band in Fig. 1E.

## Data Availability

The data that support the findings of this study are available in the Figures and the supplementary material of this article.
